# Déjà Vu in the Operating Room: Recurrent Extraction of an Anal Sexual Foreign Body

**DOI:** 10.7759/cureus.101799

**Published:** 2026-01-18

**Authors:** Isabel Sierra Fernandez, Luis Hurtado Pardo, Carla Leal Ferrandis

**Affiliations:** 1 General and Digestive Surgery Department, Hospital Arnau de Vilanova, Valencia, ESP

**Keywords:** anal emergency surgery, anal foreign body extraction, anal injuries, foreign body insertion, risky sexual behaviours

## Abstract

Rectal foreign body insertion is an uncommon presentation that may lead to serious complications if not promptly recognised and properly managed. While many cases are related to sexual experimentation, repeated insertions can reflect underlying behavioural or psychiatric conditions. The behaviour, known as polyembolokoilamania, remains insufficiently described in current medical literature, particularly when it involves recurrent episodes in the same individual. We report a 39-year-old male patient with no significant comorbidities who presented on four separate occasions over a two-year period following the insertion of rectal foreign bodies for sexual stimulation. The objects progressively increased in size, from a 5 cm spherical item to a 10 cm soft plastic ball. All were successfully removed manually under sedation, without perforation, bleeding, or other complications. Radiographic imaging was used in each episode to confirm the position and dimensions of the objects prior to extraction. This case illustrates the diagnostic and technical challenges associated with recurrent rectal foreign body insertion, as well as the need to address underlying psychosocial and psychiatric factors that contribute to recurrence. Successful management relies not only on careful surgical extraction but also on comprehensive evaluation and follow-up. A coordinated, multidisciplinary approach-combining surgical expertise with psychological and psychiatric support-is essential to prevent further episodes and to improve long-term outcomes in patients presenting with polyembolokoilamania.

## Introduction

Although uncommon, the insertion of rectal foreign bodies represents a well-recognised clinical problem that may lead to significant morbidity if not managed appropriately. The behaviour, often referred to as polyembolokoilamania, involves the deliberate introduction of objects into body orifices and is typically associated with sexual stimulation, curiosity, or underlying psychiatric conditions. Its true incidence remains underestimated due to patient reluctance to seek medical care [1-4].

This case is reported to highlight the challenges associated with recurrent rectal foreign body insertion and to allow a retrospective evaluation of management strategies. It emphasises the need to move beyond acute surgical treatment and consider structured psychiatric assessment as part of a multidisciplinary approach to reduce recurrence and improve long-term outcomes.

## Case presentation

We present the case of a 39-year-old male with no significant medical comorbidities. His medical history included surgically treated recurrent urethral stricture, uvuloplasty, and a previous episode of transverse mesocolon haematoma with hemoperitoneum managed conservatively without surgical intervention. The patient was evaluated at our institution on four separate occasions for the introduction of rectal foreign bodies for sexual stimulation.

The patient was first evaluated in 2019 after presenting with abdominal pain following the insertion of a spherical object approximately 5 cm in diameter, covered with a condom, which he was unable to retrieve. Abdominal radiography revealed a 5 cm foreign body in the distal rectum, and manual extraction was successfully performed in the operating room under sedation with the assistance of a rectoscope. In 2020, he returned after inserting a lemon that he was unable to expel. Abdominal X-ray demonstrated a rounded, non-metallic image in the upper rectum. Although urgent surgical extraction under sedation was indicated, the object was spontaneously expelled during defaecation before intervention. Later in 2020, the patient required sedation for manual extraction of a rubber ball measuring approximately 9 cm in diameter, located 6 cm from the anal verge, as confirmed by abdominal radiography. The final episode occurred in 2021, when he presented with a soft plastic ball approximately 10 cm in diameter, visualised on radiographic imaging. The object was removed manually in the operating room after deflation, without the need for additional instrumentation.

**Figure 1:**
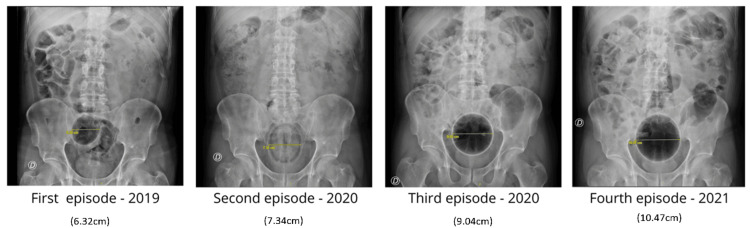
Sequential abdominal X-rays from each episode.

**Figure 2:**
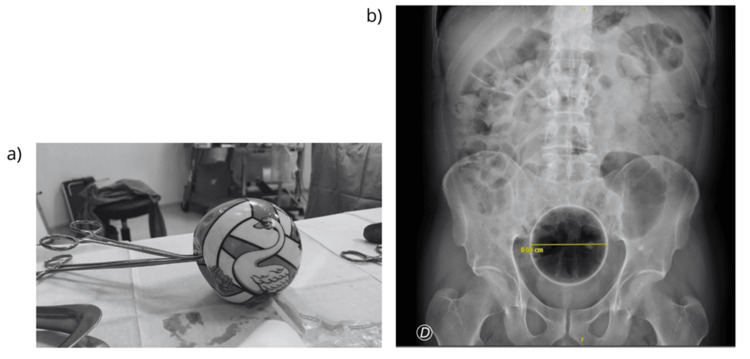
(a) Foreign body removed from the anal canal. (b) Radiological image corresponding to the foreign body (third episode).

Across these four presentations, radiographic imaging documented a progressive increase in the size of the introduced objects, ranging from 5 to 10 cm, although no complications, such as bleeding, perforation, or peritonitis, were recorded in any of the episodes (Figures 1, 2).

Postoperative recovery was uneventful after each emergency intervention, and no complications were recorded. As all presentations occurred in an emergency context and patient adherence to outpatient care was limited, comprehensive long-term follow-up and psychiatric evaluation could not be completed, leading to loss to follow-up.

## Discussion

Polyembolokoilamania-the deliberate insertion of foreign bodies through natural orifices or cutaneous openings-has been documented since the 16th century, primarily within the fields of surgery, gastroenterology, emergency medicine, urology, and psychiatry [1-3]. Although uncommon, this behaviour often constitutes a medical-surgical emergency because of the risk of infection, perforation, bleeding, and peritonitis. Despite being recognised for centuries, polyembolokoilamania remains underreported and understudied, particularly in relation to its psychiatric underpinnings [1,4].

The motivations behind this behaviour are heterogeneous, encompassing autoerotic and paraphilic practices, self-injurious behaviour, substance intoxication, psychotic episodes, developmental and obsessive-compulsive-related disorders (OCRDs), malingering, factitious disorders, and schizophrenia [1,5,6]. Recent psychiatric literature has suggested potential links between polyembolokoilamania and obsessive-compulsive spectrum disorders, with repetitive and ritualised insertion behaviour paralleling compulsive phenomena [1]. Nevertheless, detailed analyses establishing firm correlations between specific psychiatric diagnoses and object-insertion behaviours remain scarce.

Reported foreign bodies vary widely, including writing instruments, toothbrushes, batteries, bottles, glass objects, fruits, vegetables, and even animals or bones [4,6]. Sexual stimulation remains the predominant motive (≈35-40%), particularly among middle-aged males (≈86%), with no significant differences by sexual orientation [7]. In the systematic review and meta-analysis by Ploner et al. [7], sexual devices accounted for approximately 35.7% of all reported rectal foreign bodies, followed by glass (17.5%) and food items (11.2%). The estimated incidence of rectal foreign body presentation is about 0.15 cases per 100,000 inhabitants annually; however, the true figure is likely higher, given that many patients attempt self-removal and do not seek medical attention [7].

From an emergency and surgical standpoint, management of rectal foreign bodies poses notable technical challenges and is associated with significant morbidity [8,9]. Most foreign bodies can be extracted transanally under sedation or regional anaesthesia, facilitating sphincter relaxation. If the object is fragile or has sharp edges, endoanal manipulation should be avoided to prevent mucosal injury; endoscopic guidance can assist in safe retrieval [9,10]. When the object has migrated proximally to the rectosigmoid junction or cannot be grasped transanally, laparotomy may be required for distal displacement (“milking”) or direct extraction via colotomy followed by enterorrhaphy [11]. In cases complicated by large perforation, ischaemia, or diffuse peritonitis, a colostomy or Hartmann’s procedure may be indicated. For small perforations without contamination, primary closure is generally feasible [9,11]. Post-extraction evaluation with sigmoidoscopy or rectoscopy is recommended to assess mucosal integrity and rule out occult perforation [10,11].

In the present case, four separate episodes of rectal foreign body insertion were documented in the same patient, each involving progressively larger objects. This pattern suggests an escalation in behaviour, possibly linked to tolerance or increasing stimulation thresholds-a phenomenon also observed in prior reports of recurrent polyembolokoilamania [3,5,12]. Remarkably, all extractions were performed manually under sedation without major complications. Nonetheless, recurrence despite appropriate surgical management underscores that technical removal alone is insufficient; comprehensive psychiatric evaluation and follow-up are essential to prevent repetition. 

Multidisciplinary management is therefore crucial and involves colorectal surgeons, emergency physicians, psychiatrists, and psychologists. Psychiatric assessment should include screening for obsessive-compulsive spectrum and paraphilic disorders, as these have been increasingly recognised as potential contributors [1,5]. When identified, combined pharmacological and psychotherapeutic interventions can reduce recurrence and mitigate associated morbidity [1,4]. Unfortunately, long-term psychiatric follow-up data remain scarce in the literature, representing a clear research gap.

This case underscores several important clinical lessons for the management of rectal foreign body insertion. A high index of suspicion and open, non-judgmental communication are essential when assessing patients with a previous history of insertion, particularly when recurrence is suspected. Adherence to a stepwise extraction protocol, from transanal removal under sedation to laparotomy when required, helps minimise morbidity and optimise outcomes. Post-extraction endoscopic evaluation should be considered to assess mucosal integrity and to detect potential complications such as perforation or bleeding. Finally, early initiation of psychiatric and behavioural interventions, integrated into post-procedural care and maintained longitudinally, is crucial to address the underlying behavioural drivers and prevent recurrence.

## Conclusions

Recurrent rectal foreign body insertion constitutes a complex multidisciplinary challenge that extends well beyond the technical aspects of surgical management. Achieving optimal outcomes requires a coordinated approach that integrates meticulous operative care with early and sustained psychiatric intervention addressing the underlying behavioural and psychological factors. The establishment of standardised multidisciplinary care pathways may improve both immediate management and long-term prevention of recurrence. Further research is warranted to elucidate the psychopathological mechanisms driving polyembolokoilamania and to assess the efficacy of integrated behavioural and pharmacological strategies aimed at reducing relapse and enhancing patients’ quality of life.
